# The Mount Sinai cohort of large-scale genomic, transcriptomic and proteomic data in Alzheimer's disease

**DOI:** 10.1038/sdata.2018.185

**Published:** 2018-09-11

**Authors:** Minghui Wang, Noam D. Beckmann, Panos Roussos, Erming Wang, Xianxiao Zhou, Qian Wang, Chen Ming, Ryan Neff, Weiping Ma, John F. Fullard, Mads E. Hauberg, Jaroslav Bendl, Mette A. Peters, Ben Logsdon, Pei Wang, Milind Mahajan, Lara M. Mangravite, Eric B. Dammer, Duc M. Duong, James J. Lah, Nicholas T. Seyfried, Allan I. Levey, Joseph D. Buxbaum, Michelle Ehrlich, Sam Gandy, Pavel Katsel, Vahram Haroutunian, Eric Schadt, Bin Zhang

**Affiliations:** 1 Department of Genetics and Genomic Sciences, Icahn School of Medicine at Mount Sinai, One Gustave L. Levy Place, New York, NY 10029, USA.; 2 Icahn Institute of Genomics and Multiscale Biology, Icahn School of Medicine at Mount Sinai, One Gustave L. Levy Place, New York, NY 10029, USA.; 3 Department of Psychiatry, Icahn School of Medicine at Mount Sinai, One Gustave L. Levy Place, New York, NY 10029, USA.; 4 Friedman Brain Institute, Icahn School of Medicine at Mount Sinai, One Gustave L. Levy Place, New York, NY 10029, USA.; 5 Psychiatry, JJ Peters VA Medical Center, 130 West Kingsbridge Road, Bronx, NY 10468, USA.; 6 iPSYCH, The Lundbeck Foundation Initiative for Integrative Psychiatric Research, Aarhus 8000, Denmark.; 7 Department of Biomedicine, Aarhus University, Aarhus, Aarhus, 8000, Denmark.; 8 Centre for Integrative Sequencing (iSEQ), Aarhus University, Aarhus, 8000, Denmark.; 9 Sage Bionetworks, 1100 Fairview Ave N, Seattle, WA 98109, USA.; 10 Department of Biochemistry, Emory University School of Medicine, Atlanta, GA 30322, USA.; 11 Integrated Proteomics Core Facility, Emory University School of Medicine, Atlanta, GA 30322, USA.; 12 Department of Neurology, Emory University School of Medicine, Atlanta, GA 30322, USA.; 13 Center for Neurodegenerative Disease, Emory University School of Medicine, Atlanta, GA 30322, USA.; 14 Seaver Autism Center for Research and Treatment, Icahn School of Medicine at Mount Sinai, One Gustave L. Levy Place, New York, NY, 10029, USA.; 15 Department of Neurology, Icahn School of Medicine at Mount Sinai, One Gustave L Levy Place, New York NY 10029, USA.; 16 Department of Pediatrics, Icahn School of Medicine at Mount Sinai, One Gustave L Levy Place, New York, NY 10029, USA.; 17 The Alzheimer’s Disease Research Center, Icahn School of Medicine at Mount Sinai, One Gustave L Levy Place, New York, NY 10029, USA.; 18 Fishberg Department of Neuroscience, Icahn School of Medicine at Mount Sinai, One Gustave L. Levy Place, New York, NY 10029, USA.

**Keywords:** Transcriptomics, Proteomic analysis, Sequencing, Alzheimer's disease

## Abstract

Alzheimer’s disease (AD) affects half the US population over the age of 85 and is universally fatal following an average course of 10 years of progressive cognitive disability. Genetic and genome-wide association studies (GWAS) have identified about 33 risk factor genes for common, late-onset AD (LOAD), but these risk loci fail to account for the majority of affected cases and can neither provide clinically meaningful prediction of development of AD nor offer actionable mechanisms. This cohort study generated large-scale matched multi-Omics data in AD and control brains for exploring novel molecular underpinnings of AD. Specifically, we generated whole genome sequencing, whole exome sequencing, transcriptome sequencing and proteome profiling data from multiple regions of 364 postmortem control, mild cognitive impaired (MCI) and AD brains with rich clinical and pathophysiological data. All the data went through rigorous quality control. Both the raw and processed data are publicly available through the Synapse software platform.

## Background & Summary

Alzheimer’s disease (AD) affects half the US population over the age of 85 and is universally fatal following an average course of 10 years of progressive cognitive disability^1^. The certain cause of AD is known for 1-3% of early onset disease, which is autosomal dominant and 100% penetrant. Apolipoprotein E ε4 (*APOE ε4*) is a risk gene in 30% of common, late-onset AD (LOAD), but the mechanism linking *APOE ε4* to increased AD risk remains elusive^2^. Conventional genetic and genome-wide association studies (GWAS) have revealed additional loci associated with LOAD, the most recent of which is *TREM2* (refs 3–7). However, the current set of established LOAD risk loci are not informative for individual risk of developing LOAD and fail to provide actionable insights for therapeutics. Molecular profiling including whole genome, exome and RNA sequencing are key technologies that hold promise for identifying functional pathways and key targets in AD^8^. In this study, we generated whole genome sequencing, whole exome sequencing, RNA-sequencing and proteome profiling data from multiple regions of 364 postmortem control, mild cognitive impaired (MCI) and AD brains with rich clinical and pathophysiological data. All the data went through rigorous quality control. Both the raw and processed data are publicly available through the Synapse software platform.

## Methods

### MSBB AD study population

**Figure 1:**
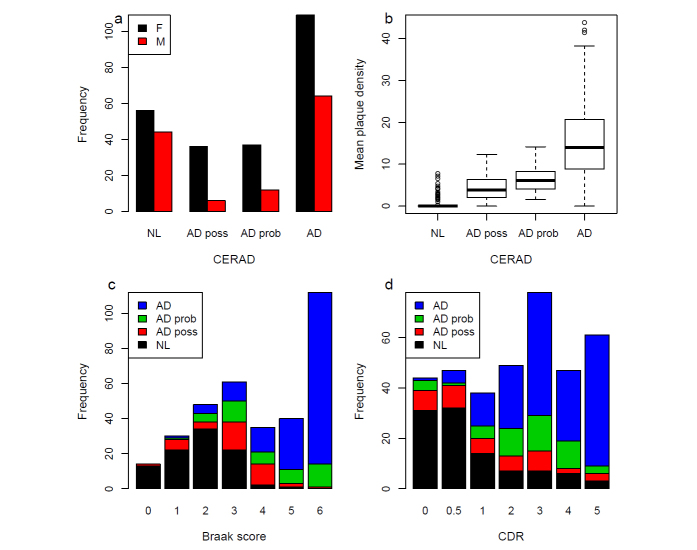
Cognitive and neuropathological trait phenotype distribution of the present study population. (**a**) Bar-chart showing the number of female (F) and male (M) samples stratified by CERAD neuropathological category. (**b**) boxplot showing distribution of mean of neuritic plaque density in cortical brain regions for each class of CERAD neuropathological categories; c and d, bar-charts showing the number of samples with different Braak score (**c**) or CDR (**d**) stratified by CERAD neuropathological category.

364 human brains were accessed from the Mount Sinai/JJ Peters VA Medical Center Brain Bank (MSBB–Mount Sinai NIH Neurobiobank) cohort, which holds over 2,040 well-characterized brains. This cohort was assembled after applying stringent inclusion/exclusion criteria and represents the full spectrum of cognitive and neuropathological disease severity in the absence of discernable non-AD neuropathology. All neuropsychological, diagnostic and autopsy protocols were approved by the Mount Sinai and JJ Peters VA Medical Center Institutional Review Boards. Neuropathological assessments, cognitive, and medical and neurological status determinations were performed according to previously published procedures as described in detail^9^. Briefly, for each sample, neuropathological assessments was performed according to the Consortium to Establish a Registry for Alzheimer's Disease (CERAD) protocol^10^ and included assessment by hematoxylin and eosin, modified Bielschowski, modified thioflavin S, and anti-β amyloid (4G8), anti-tau (AD2) and anti-ubiquitin (Dakoa Corp.). A Braak AD-staging score for progression of neurofibrillary neuropathology^11,12^ was assigned to each case. Quantitative data regarding the mean of the density of neuritic plaques in the middle frontal gyrus, orbital frontal cortex, superior temporal gyrus, inferior parietal cortex and calcarine cortex were also collected as described^9^. Clinical dementia rating scale (CDR)^13^ was conducted for assessment of dementia and cognitive status. Ages for the samples were defined as Age at the time Of Death (AOD). AOD of the present population ranged 61–108, with a mean and standard deviation (s.d.) of 84.7±9.7. Table 1 tabulates a summary of the demographic information of the present study population, including sex, AOD, mean plaque density, and Braak score, stratified by CDR score, while Fig. 1 illustrates the distribution of cognitive and neuropathological characteristics grouped by CERAD neuropathological category. There were 238 female and 126 male samples. Note that the majority (301) of the samples were of European ancestry, while 36 were African American, 25 were Latino, one was Asian, and one was unknown for race. The link to the complete demographic information of the present MSBB AD study population is provided (Data Citation 1 & Data Citation 2).

### Human brain tissue preparation

Four specific brain regions, Brodmann areas^14^, were selected for molecular profiling primarily based on our recent study of microarray profiling of 19 brain regions in AD^15^. In that study, we systematically investigated the regional vulnerability to AD at the molecular level by evaluating region-specific expression changes associated with cognitive/neuropathological traits and then rank ordered the 19 brain regions accordingly. The parahippocampal gyrus (Brodmann area 36-BM36) and the inferior frontal gyrus (BM44) were the top two most vulnerable regions and thus were selected for this study. We also chose the superior temporal gyrus (Brodmann area 22-BM22, ranked as the 7^th^ most vulnerable region) and the frontal pole (Brodmann area 10-BM10, ranked as the 14^th^) as contrast for studying the selective regional vulnerability to AD.

These four brain regions were dissected while frozen from fresh frozen, never-thawed ~8 mm thick coronal tissue blocks using a dry ice cooled reciprocating saw. The dissected regions were then pulverized to a fine powder consistency in liquid nitrogen cooled mortar and pestle and distributed into 50 mg aliquots. All aliquots were barcoded and stored at −80 ^o^C until DNA, RNA or protein isolation.

### MSBB AD RNA-seq data collection and processing

#### RNA extraction

Total RNA were isolated from brain tissues using RNeasy Lipid Tissue Mini Kit from Qiagen (cat#74804) according to the manufacturer's protocol (The RNeasy Lipid Tissue Mini Kit Handbook, Qiagen 104945, 02/2009) with these modifications: 1) all brain tissues (pulverized) were kept on dry ice before adding QIAzol Lysis Reagent, 2) tissues were suspended in the lysis reagent by vortexing with tubes placed on ice, and 3) the tissues were homogenized using a Tissue Ruptor (Qiagen, cat# 79656) at full speed for 20–30 s.

#### RNA-seq protocol

RNA-Seq library preparation was performed using the TruSeq RNA Sample Preparation Kit v2 (Illumina, San Diego, CA). Briefly, rRNA was depleted from total RNA using the Ribo-Zero rRNA Removal Kit (Human/Mouse/Rat) (Illumina, San Diego, CA) to enrich for coding RNA and long non-coding RNA. cDNA was synthesized using random hexamers, end-repaired and ligated with appropriate adaptors for sequencing. The library then underwent size selection and purification using AMPure XP beads (Beckman Coulter, Brea, CA). The appropriate Illumina recommended 6-bp bar-code bases are introduced at one end of the adaptors during PCR amplification. The size and concentration of the RNAseq libraries was measured by Bioanalyzer (Agilent, Santa Clara, CA) and Qubit fluorometry (Life Technologies, Grand Island, NY) before loading onto the sequencer. The Ribo-Zero libraries were sequenced on the Illumina HiSeq 2500 System with 100 nucleotide single end reads, according to the standard manufacturer’s protocol (Illumina, San Diego, CA).

#### RNA-seq data processing

The raw sequence reads were aligned to human genome hg19 with the star aligner (v2.3.0e). Following read alignment, featureCounts^16^ was used to quantify the gene expression at the gene level based on Ensembl gene model GRCh37.70. The gene level read counts data were normalized as counts per million (CPM) using the trimmed mean of M-values normalization (TMM) method^17^ to adjust for sequencing library size difference. The RNA-seq alignment and expression quantification matrices are available (Data Citation 3).

We also called variants in RNA-seq data by following the GATK Best Practices for variant calling on RNA-seq v3.0 (https://gatkforums.broadinstitute.org/gatk/discussion/3891/calling-variants-in-rnaseq). Starting from star aligned bam files, Picard tools were used for adding read group information, sorting, marking duplicates and indexing. Next, GATK tool SplitNCigarReads was applied to split reads into exon segments and trim any sequences overhanging into the intronic regions. Then GATK base-quality score recalibration (BQSR) was called to detect systematic errors caused by the sequencer and subsequently adjust the base quality score. After recalibration, single nucleotide polymorphisms (SNPs) and insert/deletions (INDELs) were called jointly with the GATK HaplotypeCaller. Lastly, a series of hard filters were applied to remove low quality variant sites using recommended parameters, including filtering clusters of at least 3 SNPs within a window of 35, Fisher Strand values (FS)>30.0, or Qual By Depth values (QD)<2.0.

### MSBB AD DNA whole exome sequencing (WES) and whole genome sequencing (WGS)

#### DNA isolation

DNA was isolated from 50 mg of frozen, never-thawed grey matter dissected from the frontal cortex (BM10) or superior temporal gyrus (BM22). Specimens were homogenized in 300 μl of elution buffer, and DNA was isolated using a Promega Maxwell 16 semi-automated system using the Promega Maxwell 16 Tissue DNA Purification Kit according to the manufacturer’s instructions. DNA quality and yield were assessed using an Agilent 4200 Tape Station.

#### WES protocol

Genomic DNA samples were sheared into small DNA fragments and libraries were prepared with Illumina compatible adapters and indices. Biotinylated cRNA baits were incubated with the library for 16 h and then targeted regions were selected using magnetic streptavidin beads according to Agilent SureSelect human all exon V5 kit sample preparation protocol. Targeted regions were amplified, producing an exome enriched library. The sequence ready libraries were loaded onto Illumina HiSeq 2500 System with 125 bp paired-end sequencing on V4 flow cell. Three samples were pooled per sequencing lane, aiming for 80X mean coverage per sample.

#### WES variants calling

The raw sequence reads were aligned to targeted exome regions on human genome hg19 with the BWA mem aligner. Then the sequence variants were called using the DNAseq Variant Analysis workflow of GATK Best Practices version 3, including insertion/deletion (INDEL) realignment, de-duplication, and base-quality score recalibration (BQSR). SNPs and INDELs were called jointly with the GATK HaplotypeCaller. GATK Variant Quality Score Recalibration (VQSR) was used to estimate the probability that a variant is a true genetic variant rather than a sequencing or data processing artifact. The SNP VQSR model was trained using HapMap3.3, 1KG Omni 2.5, 1KG phase1 high confidence, and dbsnp138 SNP sites, while Mills et. al. 1KG gold standard and dbsnp 138 sites were used for training INDELs model. After VQSR recalibration, a 99% sensitivity threshold was applied to filter variants by the GATK ApplyRecalibration tool. The WES alignment files and the resulting genetic variants are included (Data Citation 4).

#### WGS protocol

Whole genome sequencing (WGS) libraries were prepared at the New York Genome Center (NYGC) using the KAPA Hyper Library Preparation Kit (PCR-free) in accordance with the manufacturer’s instructions. Briefly, 650 ng of DNA was sheared using a Covaris LE220 sonicator (adaptive focused acoustics). DNA fragments underwent bead-based size selection and were subsequently end-repaired, adenylated, and ligated to Illumina sequencing adapters. Final libraries were evaluated using fluorescent-based assays including qPCR with the Universal KAPA Library Quantification Kit and Fragment Analyzer (Advanced Analytics) or BioAnalyzer (Agilent 2100). Libraries were sequenced on an Illumina HiSeq X sequencer (v2.5 chemistry) using 2×150bp cycles.

#### WGS variants calling

Paired-end 150 bp reads were aligned to the GRCh37 human reference using the Burrows-Wheeler Aligner (BWA-MEM v0.7.8) and processed using the GATK best-practices workflow that includes marking of duplicate reads using Picard tools v1.83, local realignment around indels, and base quality score recalibration (BQSR) via Genome Analysis Toolkit (GATK v3.4.0). Variant discovery was run using a two-step process. HaplotypeCaller was run on each sample individually in gVCF mode (GATKv3.4.0) producing an intermediate file format called gVCF (genomic Variant Call Format). gVCFs were combined by batches into merged gVCFs and run through a joint genotyping step (GATK v3.2.2) to produce a multi-sample VCF. Variant filtration was then performed using Variant Quality Score Recalibration (VQSR). VQSR identifies annotation profiles of variants that are likely to be real and assigns a score (VQSOD) to each variant. Variant effects annotation was then performed using SnpEff^18^, bcftools and in-house software. Other functional annotations include variant frequencies in different populations from 1000 Genomes project^19^, Exome Aggregation Consortium–ExAC, dbSNP 138^20^; cross-species conservation scores from PhyloP^21^, Genomic Evolutionary Rate Profiling (GERP)^22^, PhastCons^23^; functional prediction scores from Polyphen2^24^ and SIFT^25^; variant disease associations from OMIM, Clinvar; regulatory annotations from ENCODE^26^, Regulome^27^, KEGG pathway annotations^28^; and gene ontology annotations for biological process, cellular component, molecular function^29^. Variants and annotations were exported to tabular formats for the ease of downstream analysis. Additional filtration based on functional annotation was applied to extract variants with predicted effects on protein coding. The WGS alignment files together with the genetic variants calling and annotation are included (Data Citation 5).

### MSBB AD Proteomics

#### Tissue homogenization

Tissue samples dissected from the frontal pole (Brodmann area 10–BM10) were processed as described in ref. 30 with only minor modifications. The homogenized samples were treated with 500 μ L of urea lysis buffer (8M urea, 100 mM NaHPO4, pH 8.5), including 5 μL (100x stock) HALT protease and phosphatase inhibitor cocktail (Pierce). All homogenization was performed using a Bullet Blender (Next Advance) according to manufacturer protocols. Tissue-derived powder was added to urea lysis buffer in a 1.5 mL Rino tube (Next Advance) harboring 750 mg stainless steel beads (0.9–2 mm in diameter) and blended twice for 5 min intervals in a cold room (4 °C). Protein supernatants were transferred to 1.5 mL Eppendorf tubes and sonicated (Sonic Dismembrator, Fisher Scientific) 3 times for 5 s with 15 s intervals of rest at 30% amplitude to disrupt nucleic acids and subsequently vortexed. Protein concentration was determined by the bicinchoninic acid (BCA) method, and samples were frozen in aliquots at −80 °C. Each brain homogenate was analyzed by SDS-PAGE to assess for protein integrity as described^30^. Protein homogenates (150 μ g) were diluted with 50 mM NH_4_HCO_3_ to a final concentration of less than 2M urea and then treated with 1 mM dithiothreitol (DTT) at 25 °C for 30 min, followed by 5 mM iodoacetimide (IAA) at 25 °C for 30 min in the dark. Protein was digested with 1:100 (w/w) lysyl endopeptidase (Wako) at 25 °C for 2 h and further digested overnight with 1:50 (w/w) trypsin (Promega) at 25 °C. Resulting peptides were desalted with a Sep-Pak C18 column (Waters) and dried under vacuum.

#### LC-MS/MS analysis

Mass spectrometry analysis was performed and processed as described in ref. 30. Peptides (2 μ g) from each individual case or batch standard (BMGIS; described below) were resuspended in peptide loading buffer (0.1% formic acid, 0.03% trifluoroacetic acid, 1% acetonitrile) containing 0.2 pmol of isotopically labeled peptide calibrants (Life Technologies, #88321). Peptide mixtures were separated on a self-packed C18 (1.9 μm Dr. Maisch, Germany) fused silica column (25 cm×75 μM internal diameter (ID); New Objective, Woburn, MA) by a NanoAcquity UHPLC (Waters, Milford, FA) and monitored on a Q-Exactive Plus mass spectrometer (ThermoFisher Scientific, San Jose, CA). Elution was performed over a 120-minute gradient at a rate of 300 nl/min with buffer B ranging from 3 to 50% (buffer A: 0.1% formic acid and 5% DMSO in water, buffer B: 0.1% formic and 5% DMSO in acetonitrile). The mass spectrometer cycle was programmed to collect one full MS scan followed by 10 data dependent MS/MS scans. The MS scans (300–1800 m/z range, 1,000,000 AGC, 150 ms maximum ion time) were collected at a resolution of 70,000 at m/z 200 in profile mode and the MS/MS spectra (2 m/z isolation width, 25% collision energy, 100,000 AGC target, 50 ms maximum ion time) were acquired at a resolution of 17,500 at m/z 200. Dynamic exclusion was set to exclude previous sequenced precursor ions for 30 s within a 10 ppm window. Precursor ions with +1, and +6 or higher charge states were excluded from sequencing. A total of 7 independent batches were analyzed consisting each of 38 individual cases (randomized for age, gender, and clincopathological traits). Each batch also had the same 3 external reference standards (BMGIS, an equal mixture of all batch1 samples). These standards were run in the beginning, middle and end of each batch. From batch 7, a total of 18 individual samples and 1 BMGIS were analyzed in technical replicate and was referred to as batch 8.

#### MaxQuant for label-free quantification

RAW data for the samples were analyzed using MaxQuant v1.5.2.8 with Thermo Foundation 2.0 for RAW file reading capability. The search engine Andromeda, integrated into MaxQuant^31^, was used to build and search a concatenated target-decoy Uniprot human reference protein database (retrieved April 20, 2015; 90,411 target sequences), plus 245 contaminant proteins from the common repository of adventitious proteins (cRAP) built into MaxQuant. Methionine oxidation (+15.9949 Da), asparagine and glutamine deamidation (+0.9840 Da), and protein N-terminal acetylation (+42.0106 Da) were variable modifications (up to 5 allowed per peptide); cysteine was assigned a fixed carbamidomethyl modification (+57.0215 Da). Only fully tryptic peptides were considered with up to 2 miscleavages in the database search. A precursor mass tolerance of ±20 ppm was applied prior to mass accuracy calibration and ±4.5 ppm after internal MaxQuant calibration. Other search settings included a maximum peptide mass of 6,000 Da, a minimum peptide length of 6 residues, 0.05 Da tolerance for high resolution MS/MS scans. Co-fragmented peptide search was enabled to deconvolute multiplex spectra. The false discovery rate (FDR) for peptide spectral matches, proteins, and site decoy fraction were all set to 1 percent. Label free quantification of proteins was performed by MaxLFQ, which considered razor plus unique peptides for protein level measurements. Surrogate amyloid beta (Aβ) levels by precursor intensity trace area of tryptic fragments from the region of APP containing the Aβ sequence were quantified as described previously^30^. The MS raw data, MaxQuant parameters, search results and quantification are included (Data Citation 6).

## Data Records

All data described herein are available for use by the research community and have been deposited in the AMP-AD Knowledge Portal in study specific folders (Data Citation 1). These include sample metadata (Data Citation 2), RNA-seq alignment files in bam format and the gene level expression matrix from 4 brain regions (Data Citation 3), WES alignment files in bam format and the variants calling in plink and VCF format (Data Citation 4), WGS based variants calling in VCF format and variants annotations (Data Citation 5), and mass spectrometry raw files and protein quantification analysis results (Data Citation 6).

## Technical Validation

### WES data quality control

**Figure 2:**
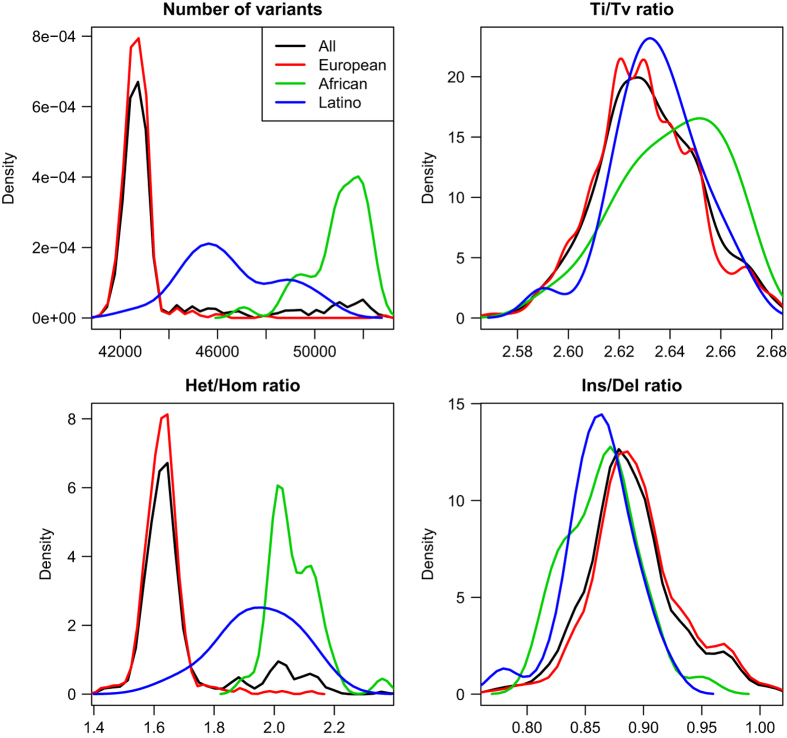
Summary of WES variants evaluation metrics. The distribution of the number of variants, Ti/Tv ratio, alternate heterozygous/homozygous (Het/Hom) ratio and indel (Ins/Del) ratio, in all samples or three major ethnic groups in the present population.

We utilized the GATK VariantEval tool to calculate various quality control metrics of the identified variants, including overall and per sample number of variants, transition/transversion (Ti/Tv) ratio, alternate allele heterozygous/homozygous (Het/Hom) ratio and insertion/deletion (Ins/Del) ratio. Overall, there were 399,135 SNPs and 22,967 INDELs identified, including 70,083 (17.6%) and 10,641 (46.3%) novel SNPs and INDELs, respectively. On average, 39,744 (364 novel) SNPs and 3,597 (228 novel) INDELs were detected per individual. The Ti/Tv ratio is a critical metric for assessing the quality of variants calling, with high quality exome sequencing variation dataset expecting to have Ti/Tv ratios between 2.8 and 3.0 (refs 32,33). Over the whole study population, the Ti/Tv ratio at the known variant sites was 2.83, close to the standard value of 2.84 in the reference dbSNP dataset. The Ti/Tv ratio at the novel variant sites was 2.12; the slightly lower ratio at new sites is consistent with previously observations^33^. Combining known and novel loci, the overall Ti/Tv ratio was 2.68. Figure 2 illustrates the per sample variant metrics. Not surprisingly, samples from different ethnic backgrounds showed different distributions in terms of the number of variants, Ti/Tv ratio and Het/Hom ratio and Ins/Del ratio. For example, the African American samples presented a much larger number of variants (mean 50974) than did the samples of European (mean 42743) or Latino ancestry (mean 46588) (one-tailed t-test P value <6.2×10^−11^). Samples of Latino ancestry presented more variants than samples of European ancestry.

### WGS data quality control

The mean mapped depth of all 349 samples was 42X (32X-62X). The paired-end short reads mapped to around 91.91% of human reference genome (GRCh37). There was no significant difference in mapped depth and breadth of coverage among the AD, MCI and control groups (Table 2). There were 32,452,033 SNPs and 5,339,491 INDELs detected in the 349 samples through a joint calling procedure. On average, each sample contained 4.14M SNPs and 264,136 INDELs (Table 2). The Ti/Tv ratio of SNPs detected was 2.08, which was within the expected range of Ti/Tv (2.0-2.1) for human whole genome. SNPeffect was further used to annotate potential functional effect of SNPs detected (Table 2).

### Proteomics data quality control and imputation

**Figure 3:**
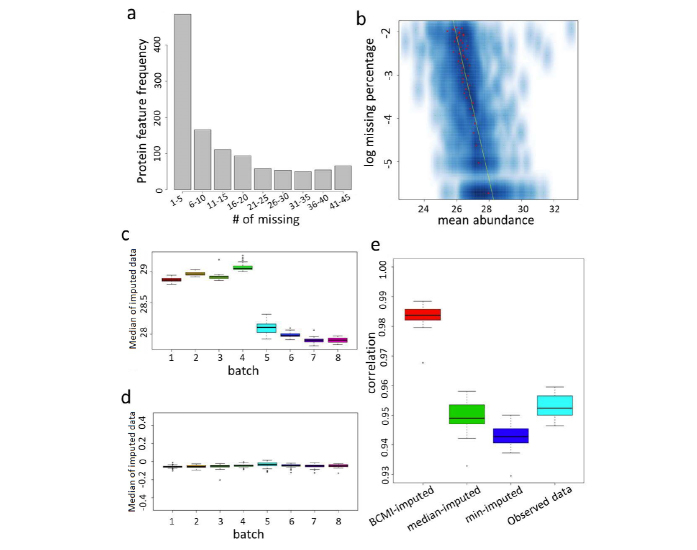
Quality check of proteomic data. (**a**) Histogram of the frequency of the proteins of each missing level. (**b**) Scatter plot of missing rate and mean abundance for each protein. The red spots indicate the cross sectional mean of each missing rate level with a yellow regression line. (**c**) Distribution of all individual median value within a batch. Panel 3a is the distribution of all batches in raw data. (**d**) The distribution of all batches in processed data. (**e**) Correlation of the duplicated samples. Red box represents the correlation of data imputed with the mixed effect model; green box represents the correlation of data imputed by observed protein median; blue box represents the correlation of data imputed by observed protein minimum; sky blue box represents the correlation of raw data.

6,107 different proteins were identified from the experiment with an average missing rate at 41.8%. By selecting proteins with missing rate smaller than 15%, we obtain protein abundance profiles for 2,962 proteins in total. Figure 3a illustrated the number of proteins with different missing percentages. In addition, these missing events were not random. Figure 3b illustrated the abundance dependent missing trend, which is rather common in mass spectrometry-based protein profiling. Moreover, due to the through-put limitation of the proteomics experiments, 306 samples were divided into 8 batches and profiled separately, which led to strong batch effects in the resulting data as samples in different batches were subjected to different experimental noise (Fig. 3c and d). Thus, it is important and necessary to remove batch effects and properly impute the missing values before any meaningful analysis can be effectively carried out. For this purpose, following Chen *et al*.^34^, we implement a mixed effects model to remove batch effects and impute missing values while taking into consideration the abundance-dependent missing mechanism in the data.

We assume that, for each protein, its abundance measurements follow a mixed effect model with a random effect term representing the batch effect. For the *k*th sample in the *i*th experiment (*i* = 1, …, *n* and *k* = 1, …, *K*) we have:

$$y_{ki}=µ+\alpha \times I_{ki}+b_{i}+e_{ki},$$

In which *y*_*ki*_ is the observed abundance, μ is the average abundance, *I*_*ki*_ is the index of reference sample taking value 1 if the *k*th sample in ith experiment is a reference sample and 0 otherwise, *α* is the fixed effect of reference samples, *b*_*i*_ is random variable with standard deviation *d* indicating the batch effect of *i*th experiment and *e*_*ki*_ is the error term representing the individual variation of the protein abundance with standard deviation *σ*_0_ of reference sample or σ of regular sample. For different *k* and *i*, *b*_*i*_s and *e*_*ki*_s are all independent. μ, α, d, σ_0_ and σ are parameters to be estimated. We also assume the abundance dependent missing data mechanism as follow:

$$P\left(M_{ki}=1\right)=\exp\left(-\gamma_{0}-\gamma_{1}\times y_{ki}\right).$$

Here *M*_*ki*_ is the missing status, taking value 1 if *Y*_*ki*_ if missing, and 0 otherwise.

With the above model, we can estimate the random effect and impute the missing values by maximizing the likelihood function of the observed data. Specifically, we implement an expectation conditional maximization (ECM) algorithm which iterates between (1) estimating the expectation of missing data and random effect conditional on the observed data and parameter estimated from last step, and (2) maximizing the conditional likelihood given the updated conditional expectation of missing data and random effect to obtain the parameter estimation, until the pre-defined convergence criterion is met.

We performed the above procedure of Batch effect Correction and Missing value Imputation (BCMI) on each protein feature separately. Notice that 18 samples have two replicates measured in both the 7th and 8th batches. We examine correlations between each replicate pair before and after the above procedure. As illustrated in Fig. 3e, replicate sample correlation increases after BCMI procedure, but decreases after the naïve imputation procedure (imputing the missing data by the median or minimum intensity of the corresponding protein), suggesting better data quality after the BCMI procedure.

### Sample matching across different datasets

In the current study, 4 different types of -omics data, including WGS, WES, RNA-seq (from 4 brain regions), and proteomics, have been generated from the same set of postmortem brain donors and brain regions, resulting in more than 1,800 samples of different data types. Quality control (QC) and assessment relating to individual data platform/technique is in place, e.g., phred-score based filtering of low quality sequencing reads and missing data imputation for proteomics. However, during the production and management of large-scale data, sample errors, including incorrect labeling, sample swapping and contamination, are inevitable but harder to detect by the platform specific QC procedure on each type of data. Because sample errors can cause both false-positive and false-negative results, systems approach for QC across multiple -omics datasets are essential for reliable and accurate identification of true biological signals from the integrative data analysis. Given the scale and complexity of such datasets, automatic QC and adjustment methods need to be applied.

**Figure 4:**
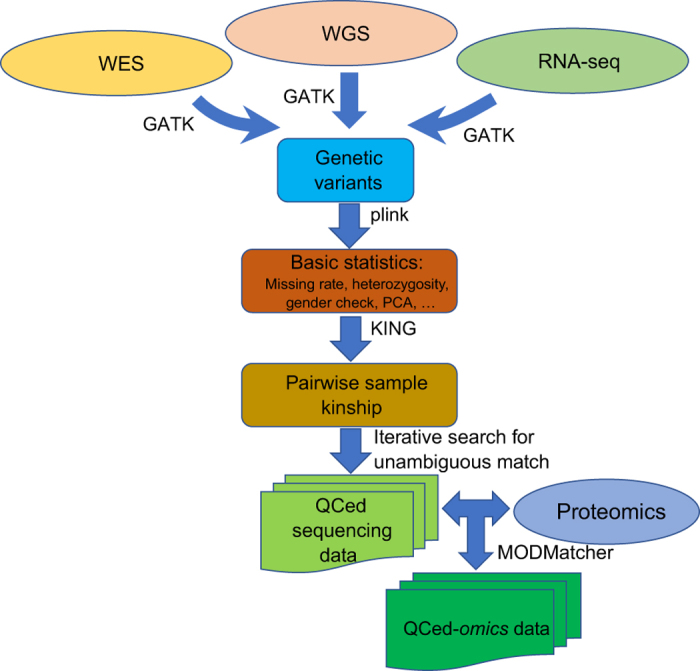
Overview of the sample alignment across data types. A robust and efficient pipeline for quality control of multiple -omics data from the MSBB AD cohort.

**Figure 5:**
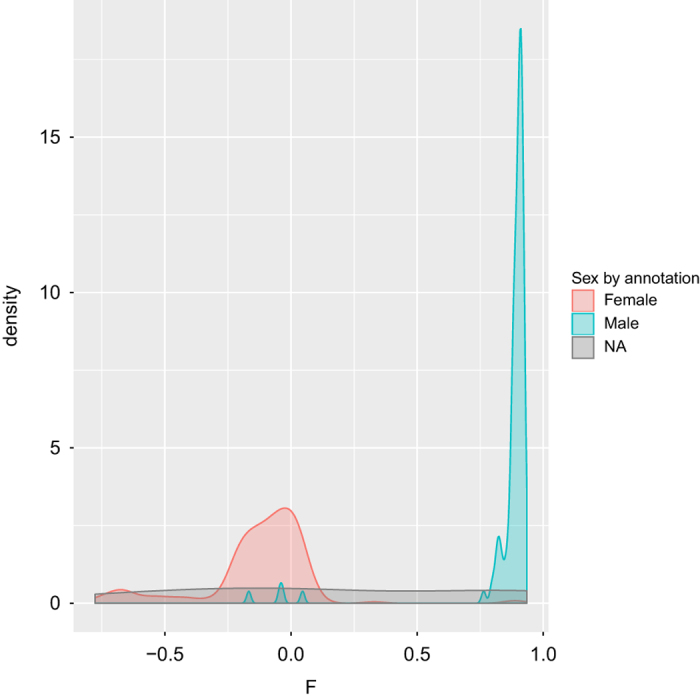
Gender imputation in the WGS data. Density showing the distribution of plink F statistic computed from X-chromosome markers. Color denotes the sex from the sample annotation table. NA, sex information not available.

**Figure 6:**
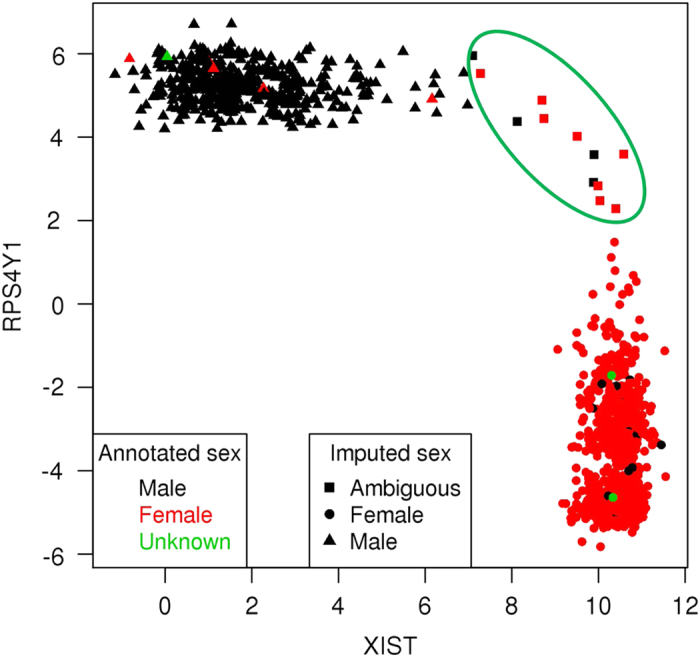
Gender imputation based on sex-specific marker gene expression in the RNA-seq data. XIST is a female specific gene, and RPS4Y1 is a male specific gene. The color denotes the sex by annotation while the shape denotes the sex by imputation. Samples highlighted in the ellipse area of the top right corner are considered ambiguous for sex imputation.

**Figure 7:**
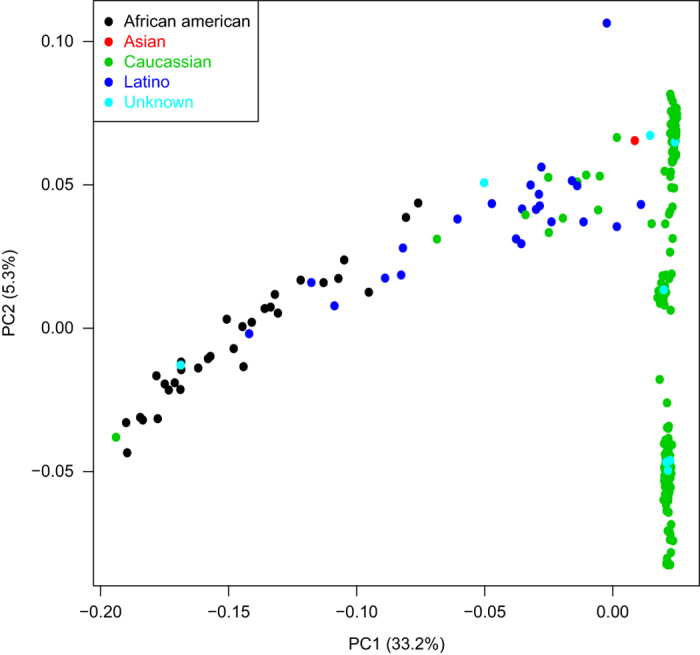
Principal component analysis suggests race-mislabeled samples in the WGS data. Scatter plot showing samples classified by the top principal components (PCs). Color denotes the race information from the sample annotation table.

Here we developed and applied a robust and efficient computational pipeline to identify consistent assignments across the multiple types of molecular data in the MSBB AD cohort. The pipeline is summarized in Fig. 4. For the various sequencing data, we first mapped the sequencing reads onto human genome GRCh37 and then called genetic variants following GATK best practices as detailed above. The genetic variants survived standard filtering by GATK were subsequently used to compute basic QC statistics including missing rate, heterozygosity, sex imputation and sample principal components analysis (PCA) using plink^35^. Variants with missing rate >50%, minor allele frequency<0.01 or Hardy-Weinberg equilibrium test P value<0.001 were removed from further analysis. Samples with excessively high or low heterozygosity rate (more than 3 standard deviations away from the mean) in each data type were marked as they suggested either mixture (possibly due to contamination) or inbreeding in these samples. Plink can impute sex from X chromosome inbreeding coefficients and flag individuals for whom the reported sex in the meta table does not match the inferred sex. It is noted that some of the statistics, including heterozygosity rate, sex imputation and PCA, require a relatively large number of independent SNPs and hence the inference is more accurate from WGS than from WES or RNA-seq. On the other hand, gender inference in RNA-seq can be facilitated by using gender-specific marker genes, such as XIST (female-specific), DDX3Y (male-specific) and RPS4Y1 (male-specific). We found 6 sex-mislabeled samples in the MSBB WGS data (Fig. 5), 20 sex-mislabeled samples in RNA-seq (Fig. 6), and 16 sex-ambiguous samples in RNA-seq. As in many genome-wide association studies (GWAS), we found top principal components (PCs) from the WGS and WES reflected population structure, in the present case, race information (Fig. 7). As a result, 4 samples were found to be mislabeled for race and 13 samples were ambiguous for race. The quality controlled (QCed) sex and race information is detailed in Supplementary Table S1.

Next we estimated pairwise sample kinship using KING^36^ and compared the genetic concordance among all sequencing samples across different data types. Since the sequencing samples from the same brain are expected to have high genetic similarity (in theory identical) while the sequencing samples from different brains are expected to present low genetic similarity, any kinship failure or mismatch suggested sample errors due to either incorrect ID labeling, sample swapping or contamination. We flagged the suspicious sample pairs which should match genetically but did not, and spurious sample pairs which should not match but present very high genetic similarity. Using an iterative procedure coupled with a majority voting scheme, we sequentially tested whether every suspicious or spurious sample was mislabeled and could be unambiguously identified with the correct brain source. Any sample which was inconclusive for its brain source was deemed problematic and discarded from further analysis. In each iteration, one sample with the most suspicious or spurious flags was selected for correction, then the suspicious and spurious flags for all samples was updated because one mislabeling may lead to multiple kinship errors and hence one correction may help resolve multiple errors.

**Figure 8:**
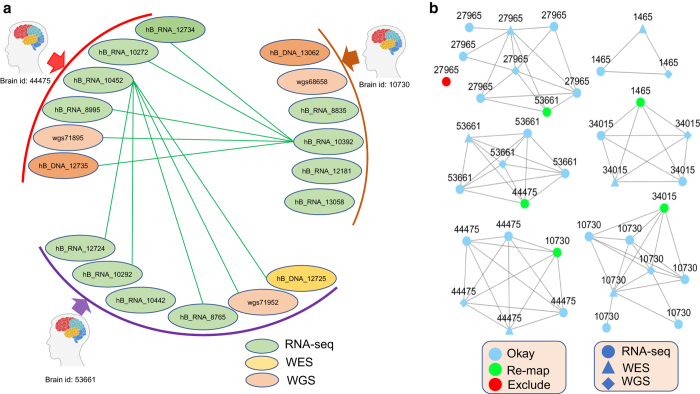
Identification of mis-labeled samples via genetic similarity test across different sequencing data types. (**a**) Sample diagram showing two mis-labeled samples. The green lines link the sample pairs inferred to be duplicate or mono-zygote (MZ) twin but associated with different brains based on sample annotation. RNA-seq samples hB_RNA_10452 and hB_RNA_10392 were considered mislabeled for their brain source. (**b)** Network illustrating the sample relationship from a subset of 6 brains. Each node denotes a sample which is labeled by the brain id based on sample annotation. Each link connects a duplicate or MZ twin pair. Light green color denotes mis-labeled samples whose brain source can be re-mapped and red color denotes a sample without genetic related pair and hence is recommended to be excluded.

**Figure 9:**
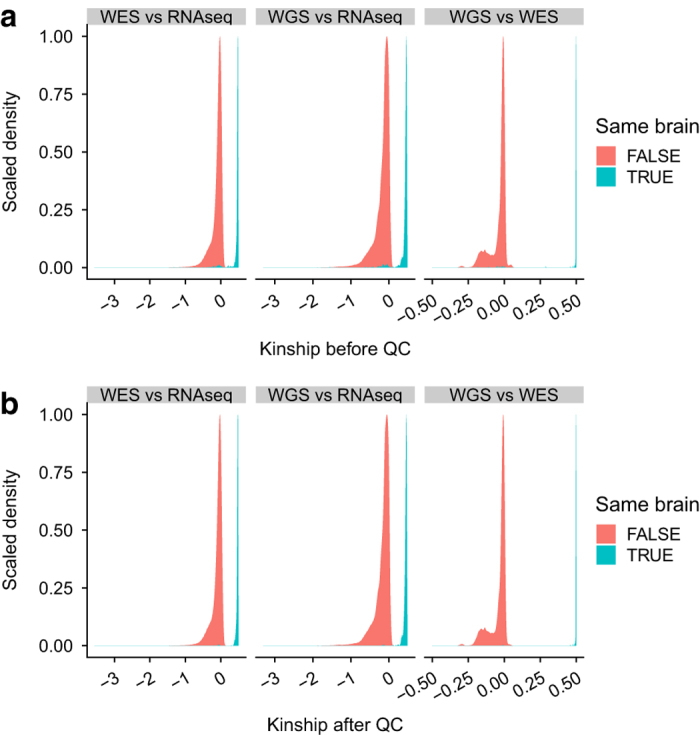
Distribution of kinship coefficients estimated between sequencing data types for samples within and between brains. (**a**) Distribution of kinship coefficients before sample QC. (**b)** Distribution of kinship coefficients after sample QC. The density has been scaled to a range between 0 and 1. Highly related (duplicate or mono-zygote twin) samples will have the estimated kinship coefficients close to 0.5, while unrelated samples will have the estimated kinship coefficients close to 0.

Figure 8a illustrates examples of mislabeled samples identified through the iterative genetic concordance analysis. There were 6 sequencing samples (4 RNA-seq, 1 WES and 1 WGS) generated from the brain 44475 according to the sample annotation. These samples showed a high within-brain genetic concordance, except for hB_RNA_10452 which exhibited a low kinship estimate (<0.1) with the other samples from the same brain, but a high kinship coefficient (> 0.4) with 5 samples from the brain 53661. According to the guideline of KING^36^, an estimated kinship coefficient >0.354 suggests a duplicate or mono-zygote twin while a kinship coefficient less than 0.1 indicates a 2nd-degree or more distant relationship. Thus, it was likely that sample hB_RNA_10452 was mis-labeled for its brain id and its true brain source should be re-mapped to 53661. Similarly, hB_RNA_10392 from the brain 10730 should be re-mapped to brain id 44475. We further identified that one RNA-seq sample, hB_RNA_10442, from the brain 53661 was also mislabeled. Resolving the true brain source for the samples in these 3 brains resulted in multiple corrections involving 3 more brains (27965, 1465 and 34015) as shown in Fig. 8b. Using the iterative genetic concordance analysis, we identified the brain identities for 10 samples (3 from RNA-seq and from 7 WGS) which were originally unknown, re-mapped the brain identities for 38 samples (29 from RNA-seq, 5 from WES, and 4 from WGS), and excluded 15 samples (8 from RNA-seq, 3 from WES, and 4 from WGS) whose brain identity was unidentifiable. The samples which were re-mapped or excluded are listed in Supplementary Table S1. Figure 9 shows the distribution of kinship coefficients estimated between 3 different sequencing data types before and after sample QC. In each plot, there are two density curves, one showing within-brain kinship coefficients with a peak close to 0.5 and the other showing between-brain kinship coefficients with a peak close to 0. It is noted that, before the sample QC, there was a small proportion of near-zero within-brain kinship coefficients (~4.5% of the pairs with kinship coefficient less than 0.1), which dramatically diminished after the sample QC (~0.36% of the pairs with kinship coefficient less than 0.1).

We used MODMatcher^37^ to match proteomics data with the QCed sequencing data. In this analysis, we considered the sample labels in QCed sequencing data as ground truth. We first computed *cis-* protein QTLs (pQTLs) based on protein expression (after missing data imputation) and WGS genotype data using MatrixEQTL and then inferred genotypes at the cis-SNPs for each proteomics sample separately. With the inferred genotypes, genetic identity similarity scores between proteomics and WGS data were computed. Second, we ran the same pQTLs based genotype imputation analysis using WES genotype data as some proteomics samples had WES genotype but not WGS genotype. In the present analysis, we considered a proteomics sample *P*_*i*_ as properly aligned if (a) the similarity score (*S*_*ii*_) with the supposed self-align WGS/WES sample *G*_*i*_ which had the same brain id was ranked in top 3 among all WGS/WES genotype profiles, or (b) *S*_*ii*_ was ranked in top 3 among all proteomics samples with respect to the genotype profile *G*_*i*_. This is slightly different from the original MODMatcher criterion which requires a reciprocal self-alignment. On the contrary, a proteomics profile would be considered mis-aligned with other unmatched genotype sample by reciprocal matching, in which we explored whether a mis-aligned genotype profile *G*_*j*_ has the highest similarity with an unmatched proteomics profile *P*_*i*_ among all proteomics samples, and the unmatched proteomics profile *P*_*i*_ has the highest similarity with *G*_*j*_ among all genotype profiles. If mislabels in proteomics data were identified, the quality of sample alignment was re-assessed according to the updated sample annotation and this process was iterated until convergence. In the present analysis, we obtained a 97.3% self-alignment rate in proteomics samples by using WGS genotype profiles and similarly a 97.1% self-alignment rate using WES genotype profiles. Collectively, we achieved a rate of 98.6% self-alignment combining the results from two genotype profiles (Supplementary Table S2). We did not observe any reciprocal mis-matched proteomics profile. We also explored a *cis* mRNA-protein mapping procedure similar to the *cis* methylation-mRNA mapping in Yoo *et al*.^37^ by computing the ranked similarity score between mRNA and protein for pairs of RNA-seq and proteomics samples. However, the self-alignment rate from this approach was only 3.6% (data not shown), which is not surprising as the correlation between mRNA and protein expression is poor (mean and median *cis* mRNA-protein Spearman’s correlations are 0.093 and 0.085, respectively).

The relatively mild correlation between the RNA-seq and proteomics data may be caused in part by the sample processing where the tissues for the transcriptomic and proteomic profilings were dissected from different locations of the same region at different times. Other factors, such as different degradation rates of RNAs and proteins in postmortem brains, may also have some impact. Nevertheless, the mild correlations between RNAs and their corresponding proteins are not unique to this cohort as we observed a similar level of mild correlations between RNAs and proteins in an independent cohort of multi-Omic data from postmortem human brains, the Mayo cohort (doi:10.7303/syn5550404), which utilized the same LC-MS/MS technique for proteomic profiling as the Mount Sinai cohort. After filtering those with more than 15% missing values in the proteomic data followed by normalization and covariates adjustment, there were 2,914 proteins remained, out of which 2,880 had matched mRNA expression data from the same brain regions in the Mayo cohort. The mean and median of the Spearman’s correlations between matched mRNA-protein were 0.116 and 0.104, respectively. Therefore, extreme caution must be taken if one wants to predict protein abundance from RNA expression, or vice versa, in postmortem brain tissues.

## Usage Notes

### Use case 1: Differential gene and protein expression in relation to AD

The present MSBB AD cohort consists of samples with varying degree of disease severity with respect to 4 different clinical and neuropathological traits in relation to AD. Stratifying samples into different severity groups allows for the differential expression of mRNAs and proteins at early or advanced stage of the disease. Investigators can prioritize genes which are altered in both mRNA and protein levels. Moreover, RNA-seq based mRNA expression was profiled in 4 different brain regions which can help identify region-specific gene signatures implicated in selective vulnerability to AD^15^.

### Use case 2: Identification of gene and protein expression quantitative loci and novel disease associated genetic variants

Genome-wide association studies (GWAS) have successfully identified more than 30 common genetic variants associate with the disease risk of late onset AD. However, the majority (>75%) of the phenotypic variance remain unexplained by the known markers^38^. Massive genome sequencing from the present WGS and WES data offers an unprecedented opportunity to characterize both low frequency and rare variants that may have a profound impact on disease risk but otherwise missed by traditional GWAS. However, we must notice that the sample size is small and hence the power is very limited.

In addition, a joint analysis of WGS/WES genotype, RNA-seq and proteomics profiles can identify variants playing transcriptional or translational regulatory roles by the so-called expression and protein quantitative trait loci (eQTLs and pQTLs) analysis. Together with the genotype-disease association from this cohort and other public datasets, this could help identify variants conferring risk to AD via transcriptional or translational regulatory mechanisms.

### Use case 3: Gene and protein integrative network analysis

AD is a complex human disease with a multifactorial nature involving a system of genetic and environmental interactions. Thus, there is a growing interest in applying an integrative network biology approach to model the interconnected relationships among a large network of gene/protein expression traits, genetic variants and clinical/neuropathological outcomes^39^. The network analysis approaches (e.g. WGCNA^40^, MEGENA^41^, Bayesian causal networks^42^ and other similar algorithms) combined with the deep and comprehensive multiscale -omics data can not only help identify gene/protein modules that were enhanced or disrupted in AD but also prioritize network hub regulators driving the key biological process implicated in AD.

## Additional information

**How to cite this article**: Wang, M. *et al*. The Mount Sinai cohort of large-scale genomic, transcriptomic and proteomic data in Alzheimer's disease. *Sci. Data* 5:180185 doi: 10.1038/sdata.2018.185 (2018).

**Publisher’s note**: Springer Nature remains neutral with regard to jurisdictional claims in published maps and institutional affiliations.

## Supplementary Material



Supplementary Table 1

Supplementary Table 2

## Figures and Tables

**Table 1:** Demographics for the study population stratified by CDR.

**CDR**	**Total size**	**Female/Male**	**Age of death**	**Mean plaque density**	**Braak score**
0	44	32/12	82.4±9.4	2.1±2.9	2.1±1.3
0.5	47	24/23	81.7±11.4	3.2±5.2	2.3±1.5
1	38	26/12	84.9±10.6	6.3±6.3	3±1.8
2	49	31/18	86.5±7.7	9±8.5	4.5±1.7
3	78	56/22	86.4±8.5	9.8±7.8	4.7±1.5
4	47	30/17	87.7±7.8	10.6±7.7	4.6±1.6
5	61	39/22	82.3±10.8	18.6±11.4	5.1±1.3

**Table 2:** Summary of the whole genome sequencing data.

**Group**	**Total**	**AD**	**MCI**	**Control**	**Unclassified**
Sample number	349	258	43	40	8
Total raw bases (Gb)	47,234.85	35,000.30	5,781.90	5,344.65	1,108.00
Total mapped bases (Gb)	46,905.02	34,759.08	5,738.92	5,306.18	1,100.84
Mean raw bases per individual (Gb)	135.34	135.66	134.46	133.62	138.5
Mean mapped bases per individual (Gb)	134.4	134.73	133.46	132.65	137.61
Mean mapped depth (X)	42.56	42.67	42.26	42	43.58
breadth of coverage (% of genome)	91.91	91.902	92.02	91.85	91.92
Mean read length	151	151	151	151	151
No. of SNPs	32,452,033	28,279,155	17,291,042	15,350,134	7,854,397
bi-allelic	31,714,836	27,648,823	16,979,800	15,081,052	7,773,748
multi-allelic	737,197	630,332	311,242	269,082	80,649
Mean variant SNP sites per individual	4,138,872	4,126,627	4,225,036	4,138,235	4,053,271
Ti/Tv ratio	2.08	2.08	2.08	2.08	2.08
Indels	5,339,491	4,621,397	2,569,428	2,225,159	936,046
Mean variant Indel sites per individual	264,136	262,706	273,735	265,023	254,236
3-prime UTR variant	226,130	193,011	112,299	98,632	48,104
5-prime UTR premature start codon gain variant	8,201	6,917	3,785	3,303	1,508
5-prime UTR variant	43,102	36,417	20,741	18,279	8,727
initiator codon variant	26	20	10	13	6
intergenic region	12,968,846	11,190,326	6,909,837	6,158,825	3,186,627
intragenic variant	1,720,234	1,479,298	905,608	803,650	408,297
intron variant	12,273,865	10,521,791	6,335,143	5,592,120	2,802,259
missense variant	151,628	127,117	65,494	58,348	26,720
missense variant&splice region variant	3,525	2,919	1,467	1,286	564
non coding exon variant	140,928	123,097	77,928	69,429	37,377
protein protein contact	811	674	304	252	92
splice acceptor variant&intron variant	1,178	990	521	466	246
splice acceptor variant&splice donor variant&intron variant	15	9	7	5	4
splice acceptor variant&splice region variant&intron variant	11	8	6	3	1
splice donor variant&intron variant	1,622	1,383	771	689	355
splice donor variant&splice region variant&intron variant	9	5	3	2	0
splice region variant	995	853	505	441	205
splice region variant&intron variant	22,085	18,973	11,190	9,943	4,915
splice region variant&non coding exon variant	4,027	3,506	2,163	1,976	1,029
splice region variant&stop retained variant	6	5	2	3	1
splice region variant&synonymous variant	2,729	2,342	1,314	1,173	572
start lost	231	197	92	81	33
start lost&splice region variant	7	5	2	1	0
stop gained	2,595	2,165	911	787	340
stop gained&splice region variant	73	57	24	21	7
stop lost	158	137	84	74	46
stop lost&splice region variant	20	19	13	13	8
stop retained variant	80	70	47	42	32
synonymous variant	115,128	99,186	57,983	51,292	25,120
upstream gene variant	2,991,606	2,576,772	1,586,065	1,410,548	733,828
downstream gene variant	2,395,694	2,062,423	1,268,135	1,127,516	583,990
